# 4441 Comparing 3 methods of assessment of psoas area as a proxy for sarcopenia in predicting short-term outcomes in trauma patients 55 years and older

**DOI:** 10.1017/cts.2020.376

**Published:** 2020-07-29

**Authors:** Anna Meader, Mihaela Stefan, David E. Clark, Christine W. Lary, Paul K. J. Han

**Affiliations:** 1 Tufts University; 2 Baystate Medical Center; 3 Maine Medical Center Research Institute

## Abstract

OBJECTIVES/GOALS: **Specific Aim 1** To examine sex distribution of psoas cross sectional area (CSA) on CT imaging in a cohort of trauma patients age 55 and older. We will use three methods of assessing psoas CSA: psoas CSA averaged between left and right, average psoas CSA adjusted for height, and average psoas CSA adjusted for body surface area (psoas index). **Specific Aim 2** Use multivariable logistic regression prediction modeling to compare the 3 methods of CT psoas muscle measurement widely used in the literature in their ability to predict a composite of in-hospital morbidity and mortality in trauma patients ages 55 and older. METHODS/STUDY POPULATION: The Maine Medical Center Trauma Registry is maintained by the Trauma Surgery Service at Maine Medical Center in Portland, Maine, the only Level-1 trauma center in the state. After receiving approval from the Institutional Review Board of Maine Medical Center for this retrospective cohort study, we queried the Maine Medical Center Trauma Registry for all adults 55 years and older who underwent evaluation by the Trauma Service between January 1, 2015 and January 1, 2019. In the case of multiple admissions within the study time period, only a patient’s index admission was used. MaineHealth IMPACS imaging software was used to measure bilateral psoas CSA on each patient CT. The Maine Medical Center electronic medical record was queried for additional clinical information including the ICD codes associated with each patient encounter. Data analysis was performed using R statistical software (R project, Vienna, Austria). Data is reported as median + IQR for CSA measurements. The agreement between the three methods of quantifying psoas CSA was evaluated using Pearson correlation (R package “stats”). Inter-rater reliability of psoas muscle measurements was evaluated using intra-class correlation (R package “irr”). Prediction models for the composite outcome of in-hospital morbidity and mortality were constructed using multivariable logistic regression. Bootstrapping was used for internal validation and shrinkage to avoid overfitting. Models including psoas CSA were compared to a baseline model without psoas CSA to evaluated incremental added predictive ability. RESULTS/ANTICIPATED RESULTS: This cohort provides a basis for examining the population distribution of psoas CSA in adults 55 years and older. IN addition to a high level of agreement between the three methods of measuring psoas CSA (Spearman coefficient > 0.9), there was also high level of inter rater reliability in psoas muscle assessment (intraclass correlation 0.9). We anticipate that psoas CSA adjusted for body surface area will add the most incremental predictive ability to a model predicting in-hospital morbidity and mortality. DISCUSSION/SIGNIFICANCE OF IMPACT: Given the heterogeneity of health status amongst elderly trauma patients, a major challenge lies in the rapid objective identification of those elderly trauma patients who are frail. Due to the limitations in current frailty measures, there has been a surge of interest in surrogate markers of frailty, such as muscle mass, as predictive factors of poor outcomes after trauma.Several studies have found that sarcopenia is associated with post injury morbidity and mortality. Estimates of the prevalence of sarcopenia among trauma patients vary across studies due to differences in definition and sample characteristics. In order to appropriately categorize patients as sarcopenic, the population distribution of psoas CSA on CT must be established. The psoas measurement that best correlates with outcomes has yet to be determined, and it is unclear which measurement should be implemented in usual practice. Our main objective is to improve the outcomes of sarcopenic patients hospitalized with trauma by implementing in the future patient-centered interventions which will account for sarcopenia.

